# Constructing a Bioethical Framework to Evaluate and Optimise Newborn Bloodspot Screening for Cystic Fibrosis

**DOI:** 10.3390/ijns6020040

**Published:** 2020-05-26

**Authors:** Rachael E. Armstrong, Lucy Frith, Fiona M. Ulph, Kevin W. Southern

**Affiliations:** 1 Department of Women’s and Children’s Health, University of Liverpool, Liverpool L12 2AP, UK; r.armstrong3@doctors.org.uk; 2 Institute of Population Health, University of Liverpool, Liverpool L69 3GL, UK; frith@liverpool.ac.uk; 3 Division of Psychology & Mental Health, School of Health Sciences, Faculty of Biology, Medicine and Health, University of Manchester, Manchester Academic Health Science Centre, Manchester M13 9PL, UK; Fiona.Ulph@manchester.ac.uk

**Keywords:** cystic fibrosis, newborn bloodspot screening, cystic fibrosis screen positive, inconclusive diagnosis (CFSPID), bioethics

## Abstract

Newborn bloodspot screening for cystic fibrosis is a valid public health strategy for populations with a high incidence of this inherited condition. There are a wide variety of approaches to screening and in this paper, we propose that a bioethical framework is required to determine the most appropriate screening protocol for a population. This framework depends on the detailed evaluation of the ethical consequences of all screening outcomes and placing these in the context of the genetic profile of the population screened, the geography of the region and the healthcare resources available.

## 1. Introduction

In this paper, we develop a bioethical framework that can be used to assess newborn bloodspot screening (NBS) for cystic fibrosis (CF) protocols [1,2]. This approach provides a means to appraise the impact of the evolving technologies that are increasingly being used as part of screening protocols, for example more extensive DNA analyses [3]. An ethical consideration is essential for all screening strategies but particularly pertinent for NBS for CF for three main reasons: (1) NBS for CF protocols are complex and varied, (2) expanded DNA analysis including sequencing is increasingly common and (3) outcomes are complicated and not comprehensively recognised or characterised.

The measurement of immuno-reactive trypsinogen (IRT) from a dried bloodspot (DBS) sample enabled screening for CF by providing a simple and inexpensive test, as per the criteria outlined by the World Health Organisation (WHO) [4]. However, other criteria outlined by Wilson and Jungner were more challenging to fulfil, namely that an early diagnosis following a positive NBS resulted in improved outcomes for children with CF. This felt at odds with the lived experience of regions in which NBS for CF was established, when qualitatively the impact of an earlier diagnosis seemed considerable [5].

In an important study undertaken in Wisconsin (USA), over 650,000 DBS samples were analysed for IRT (and in some cases DNA analysis), but only results from alternative samples were reported [6]. In 54 of the samples reported, a positive NBS result was associated with an early diagnosis and significantly improved nutritional outcomes, compared to 67 infants diagnosed clinically. These early differences in nutritional well-being were transient, reflecting the capacity of standard care to address the early challenges of growth delay in clinically diagnosed children [7]. There were no clinically significant differences in respiratory outcomes, and this was consistent with a study undertaken in the United Kingdom [8,9].

**Figure 1:**
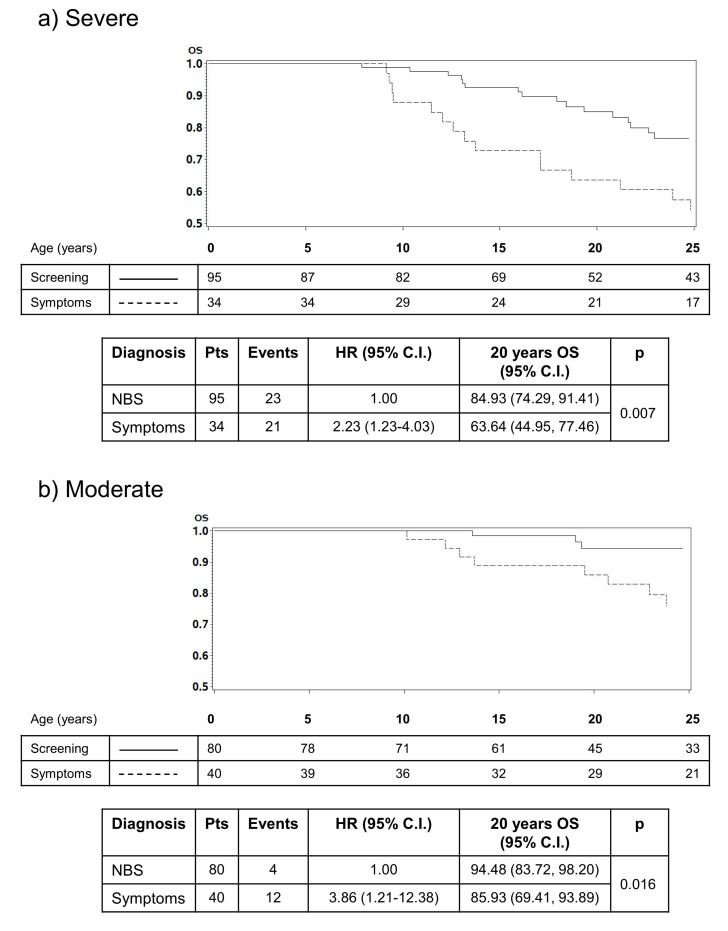

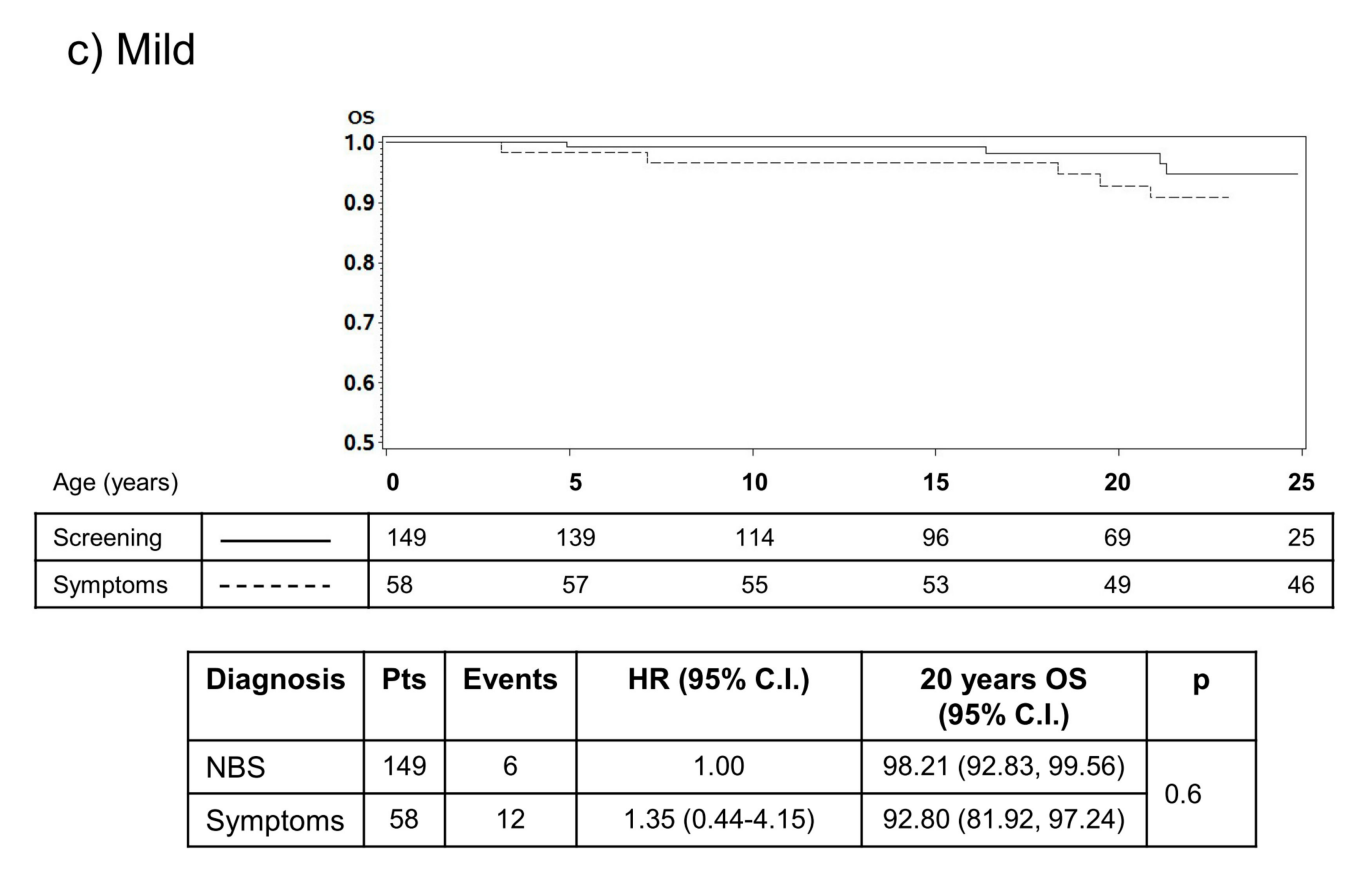
Results from an Italian study that illustrates the impact of early diagnosis through NBS on survival. The impact on survival is most apparent in infants with severe (**a**) or moderate disease (**b**) after diagnosis, highlighting the reduced impact of early diagnosis on people with mild or less typical CF (**c**) [12].

For those who work in the field, there was a disconnection between the apparent positive impact of NBS in the clinical environment and the relative lack of evidence for improved outcomes for people with CF after NBS. To some degree, this reflects the ability of CF teams and parents to provide high quality CF care following a clinical diagnosis, even if delayed [9]. However, evidence is increasing that small differences in clinical outcomes in the early months of life can lead to significant and sometimes dramatic differences in outcomes later in life. This is supported by the historical cohort study undertaken in Australia that compared two groups of children with CF before and after the implementation of NBS for CF [10]. Over the first two decades, differences between the two cohorts were minimal, but a distinct and large difference in survival became apparent as the cohorts entered their adult years, with the clinically diagnosed group faring much less well. These data are supported by data from other programmes, including an Italian group reinforcing the view that early recognition and treatment through NBS may have a more profound impact on clinical outcomes later in life [11,12] (Figure 1).

There are other important but “softer” outcomes that support the rationale of NBS for CF—namely, preventing the diagnostic odyssey that many families have to endure, providing families with information to inform future reproductive decisions and providing a framework for improved and more systematic CF services to be established across a region [1].

On the basis of these data, there is a compelling rationale to support NBS for CF, but also a responsibility to ensure that protocols are designed to minimise negative outcomes [1]. It is clear that the WHO screening criteria alone are not sufficient to critically judge the validity of a programme. Petros suggests an expanded list of criteria which provides a more comprehensive picture of the worth of an NBS programme [13]. Using these criteria, he argues that NBS for CF is a valid and worthwhile undertaking. These additional criteria are helpful, but still not adequate to rigorously evaluate a programme in the era of new emerging technologies, especially the ability to undertake extensive DNA testing in a cheap and timely manner. Advances in the capability to undertake extensive genetic testing have outpaced the capacity of most to assimilate this knowledge, and for many it is a potential cause of significant anxiety [14]. In such a climate, we propose that a bioethical approach is required to provide a framework to assess and optimise NBS for CF to ensure that the most appropriate protocol is used for a given population.

To work towards a bioethical framework, it is important to consider the impact of all possible outcomes from NBS for CF both in isolation and collectively, weighing up the relative importance to society and individuals. To establish a framework, we examined all potential outcomes for NBS for CF and related these to ethical considerations.

This work was undertaken in a series of small group meetings between the author team, with subsequent email correspondence and a literature review.

## 2. Outcomes from Newborn Bloodspot Screening for CF

Below, we list the potential outcomes from NBS for CF and the bioethical issues raised by each.

### 2.1. True Negative: An Infant with a Negative NBS Result, Not Affected by CF

An effective outcome of the screening programme is the accurate exclusion of cystic fibrosis. The accurate identification of infants who do not have CF is a positive outcome with no associated adverse consequences. As with all screening, this societal benefit is accepted and often underappreciated by the general population.

### 2.2. True Positive: An Infant with CF Detected by NBS

Early diagnosis provides a window of opportunity for proactive nutritional intervention. The impact on well-being later in life is now becoming apparent, as outlined in the introduction.

The beneficial implications of a true positive NBS result are:

- Proactive treatment.
- An earlier diagnostic period provides an opportunity to initiate an earlier referral into specialist tertiary care centres and to start positive intervention at the earliest opportunity.
- Preventing a potentially protracted and painful diagnostic journey.
- Diagnostic delays for children affected by cystic fibrosis have been shown to be associated with negative psychosocial consequences [15]. The journey to a clinical diagnosis may be protracted and more complicated, with higher levels of psychological distress for the individual and family. The early detection of CF in a newborn prevents this by providing a timely diagnosis and access to the appropriate specialist CF team [16].
- Information for reproductive decision making.
- A positive NBS result, subsequently confirmed by diagnostic assessment, provides an opportunity for the parents (and other family members) to obtain further information about the implications of CF and to have the option to consider future reproductive decisions. The parents should be provided with genetic counselling to guide them through this process, mitigate unaddressed fears and uncertainty and enable them to make informed decisions.

A potential negative impact of a true positive NBS result is the possibility of revealing non-paternity (for example, an infant is recognised to be homozygous for F508del, but subsequent genetic testing of the parents demonstrates that the father is not a carrier). Although this is a rare negative compared to the considerable positive impact, it is important that appropriate follow up is in place for parents to seek genetic counselling and support following the NBS process, and this should be empathetic and sensitive.

Whilst a true positive result is of benefit to the infant and family, this needs to be balanced with the potential for harm. Parents/carers may consider that they have had time with a “normal” child diminished, and the initial processing of a positive result needs to be handled sensitively, with a particular focus on timeliness and clear information. It is important that this situation is not presented to families as a “good news story” that their child has been diagnosed early, and neither should it be presented dramatically. Rather, it should be presented as the start of a journey to a hopefully full and active life. The communication of the result and the subsequent diagnostic assessment is a task requiring experience and skill, and some parents are particularly vulnerable at this point. Healthcare professionals should be able to recognise this and address the parents’ needs appropriately over time.

It is essential that a newly diagnosed infant with CF has access to high quality CF care which does not place them at risk (for example, from cross infection with airway pathogens). Health economic analysis demonstrates that whilst infants with CF recognised through NBS may have similar outcomes to those diagnosed clinically, this is at the expense of less treatment intervention (i.e., clinically diagnosed infants need more treatment to achieve similar outcomes) [17]. Hence, a societal benefit is achieved. It is clear that from a bioethical view, a true positive result achieves considerable individual and societal benefit but occasional harm; programmes must strive to minimise harm (non-maleficence). This can be done through the effective and timely processing of the NBS result, effective communication and a clear pathway to high quality multi-disciplinary care.

### 2.3. Recognition of an Infant as a Carrier (Negative NBS Result)

For some programmes (for example, The Netherlands and UK), carrier reporting is a possible outcome. For most programmes using DNA analysis as a second tier of testing, carriers are only confirmed after clinical assessment and sweat testing. For these families, this result would be consistent with a false positive experience (see next section). Carrier reporting may leave the family with some anxiety around the possibility of an unrecognised second Cystic Fibrosis Transmembrane Conductance Regulator (CFTR) variant, and clear information is required to minimise this potential harm.

The detection of carrier status in a baby provides an opportunity for the parents to undergo genetic counselling and testing, which may impact on future reproductive decision making and cascade screening for other family members. There is a potential for negative impact arising from carrier recognition. There is evidence of increased parental anxiety about the child; in one study, a significant number of parents (18%) felt anxious about the health of their child four years after screening, suggesting a lack of assimilation of the “healthy carrier” concept [18]. The delivery and communication of carrier status are key factors, with most concern being caused by uncertainty or confusion regarding the use of negative terms such as “query” around the screening result [19]. A perceived negative style of communication from the healthcare professional can cause increased anxiety, and lead to family issues (stigma), depression and relationship difficulties [20]. Indeed, this study suggested that parents do not perceive the information that their child is a carrier as negative, rather, the distress arises from communication practices. Concerns for the family include anxiety about the status of their other children and how to communicate the result with other family members [21]. This work found that whilst some families could be very supportive, other families could find this communication very challenging and may effectively “shut down” conversations, blame the person starting the conversation, or increase existing family stress. Providing effective support for people could lead to families benefiting from the information rather than it becoming a disruptive factor.

As with a true positive result, carrier recognition is associated with the potential risk of recognising non-paternity. This needs to be considered in the processing of a carrier result, for example leaving further genetic counselling as an option to the parents rather than making it compulsory, as in some programmes. 

An ethical issue raised by all newborn screening is the possibility of reducing autonomy, as those screened have no opportunity for consent or control over the data generated. Self-determination does not feature in these public health screening strategies. This is balanced against the rights of children to receive optimal healthcare. Carrier recognition throws this into sharp relief, and the ownership of this information requires careful consideration. It is imperative that young adults identified as carriers through NBS receive this information in a clear and sensitive manner. At present, there is no clear guidance for programmes on this issue on what information and in what format to pass on [22]. It is assumed to be the responsibility of the adults who receive the initial result (parent/carers and primary healthcare professionals), who may not appreciate or want to pass on the information. As there is no clear guidance on how to manage this situation, there is a potential for harm when the index case becomes aware of their carrier status or if they are denied information on which to make fully informed decisions about their reproductive options.

### 2.4. False Positive: An Infant with a Positive NBS Result, Who Does Not Have CF

False positive NBS results are associated with acute psychological distress for the parent/carers, and for some this may extend into longer-term psychological morbidity [23]. The period of time between a false positive NBS result and the subsequent diagnostic testing has been shown to be acutely stressful for the family [24]. Qualitative research has shown that parents experience feelings of guilt for having passed on a “faulty” gene, and that even following confirmative diagnostic testing to exclude CF, they still harbour feelings of scepticism and fear that the test may be wrong, with subsequent anxiety for their child’s health. This may affect how parents perceive and respond to healthcare services in the future, and may undermine their confidence in medical services [23,25].

A population-based cohort study reported higher maternal worry scores for mothers with an infant with a false positive NBS result and, even following adjustment for confounding variables, there was a higher proportion of parents using outpatient and accident and emergency services for infants with a false positive NBS result compared to matched controls [26]. Care should be taken to ensure parents are fully informed in the antenatal period about the screening process and subsequent diagnostic testing, in order to avoid misconceptions about the screening result and prevent psychosocial confusion and the over-medicalisation of healthy infants [19]. In one prospective study, 96% of parents expressed having been anxious at the time of being informed about the need for a diagnostic sweat test, and the anxiety increased proportionally to the waiting time for diagnostic testing; 86% felt entirely reassured three months after the test. This suggests that the distress associated with a false positive result is most likely to be temporary [27]. Participants in a qualitative study have commented that they would be willing to endure the acute psychosocial risks of a false positive result in acknowledgement of the potential value of a true positive result and the recognition of the health benefits of an early diagnosis [23,26].

Families who remained anxious 3 months after diagnostic testing described doubts regarding the test’s reliability, a poor understanding of the screening and diagnostic process and fears of CF being transmitted to future generations [27]. To minimise undermining parental confidence in the healthcare system, it is important to counsel families about the screening process, particularly when the initial result requires further diagnostic confirmation; this is particularly important when the initial screening result is positive, as there is an increased potential for misconceptions and doubt about the reliability of further testing. This information and support needs to be provided antenatally and be reinforced throughout the pregnancy, birth and postnatal period.

A small subset of parents continued to have persisting concerns about the health implications for their child following a false positive NBS result; namely, concerns of the possibility of poor health and illness for the infant, the fear of CF affecting another child or a genetic mutation affecting another family member, with some cases leading to a ‘finger pointing’ culture of attempting to identify the source of a suspected mutation [23]. It is important that screening programmes incorporate a recognition of the importance of counselling families on the meaning of a false positive NBS result, and that parents have the opportunity to express and discuss their concerns, with access to appropriate genetic counselling services where required.

Although, in general, the distress associated with a false positive result is considered transient, it may be that there are subtle longer-term psychosocial issues that quantitative studies may not have been sensitive enough to identify. Regardless, there is good agreement that NBS for all conditions should aim to minimise false positives. The European CF Society (ECFS) standards of care document suggests that CF programmes should aim for a positive predictive value greater than 0.3 (i.e., for every 10 infants with a positive NBS result, more than three will have a confirmed diagnosis of CF) [28]. 

### 2.5. Unresolved Cases

In some cases, a positive NBS result may remain unresolved. For example, an extremely preterm infant may have a positive NBS result, with a raised IRT level and one CF-causing gene variant detected. The infant may die from complications of preterm birth before further diagnostic testing is possible. Although this is a rare event in screening programmes, the increased incidence of raised IRT measurements in sick infants and infants with profound chromosomal abnormalities makes this a situation that occurs more frequently than might be anticipated by chance [29].

An unresolved screening outcome may cause psychological morbidity for the affected parents. The significance of the screening result is unknown, potentially causing increased concerns for future reproductive decisions. In addition, this result is presented during a period of extreme distress for parents, who may be unable to assimilate the information correctly. 

Unresolved anxieties and a lack of diagnostic certainty may contribute to longer-term psychological distress, particularly in the context of further reproductive decisions in the face of uncertain genetic information.

### 2.6. Cystic Fibrosis Screen Positive, Inconclusive Diagnosis (CFSPID)

A potential outcome from a positive NBS result is an inconclusive diagnosis [30,31]. The number of infants with an unclear diagnosis is dependent on the NBS approach taken, with increased recognition associated with increased DNA analysis [32]. For example, a protocol that employs extended gene sequencing will recognise more infants with an unclear diagnosis, and these infants will be more likely to remain well. Biochemical protocols with no DNA analysis will still recognise infants with an unclear diagnosis but at a much lower rate and with intermediate sweat test results (a sweat chloride concentration of between 30 and 59 mmol/L). Infants with an intermediate sweat chloride are more likely to develop clinical features consistent with CF and convert to a CF diagnosis [33].

A number of papers have considered the evaluation and management of these infants and this is covered in another chapter in this series, as are the issues around designation [34]. Recently, a global exercise resulted in the harmonisation of the definition and designation of these infants as Cystic Fibrosis Transmembrane Conductance Regulator (CFTR)-Related Metabolic Syndrome/Cystic Fibrosis Screen Positive, Inconclusive Diagnosis (CRMS/CFSPID) [30,31]. For this paper, we will use the term CFSPID.

Infants with a CFSPID designation are by definition healthy, and the majority will remain healthy through their childhood. There are three pathways that can lead to a conversion to a CF diagnosis:

1. A repeat sweat test becomes supportive of a CF diagnosis (sweat chloride >59 mmol/L).
2. New information emerges that the CFTR variant is CF-causing (two CF-causing variants equates to a CF diagnosis, regardless of the sweat chloride value).
3. The CFSPID infant develops symptoms consistent with a CF diagnosis (most commonly a chronic cough).

For a programme using extended gene sequencing, around 5% of the infants designated as CFSPID will convert to a CF diagnosis [25]. For programmes with limited or no DNA analysis, conversion is more common (between 25% and 40%), although total numbers will be less [33]. If a programme that employs extended gene sequencing only reports variants that are CF-causing, this will significantly reduce the recognition of infants with CFSPID.

The majority of infants who do not convert to a CF diagnosis face an increased risk of a CFTR-related disorder (CFTR-RD) later in their life. A CFTR-RD is defined as a condition (usually single organ) that is associated with abnormal CFTR function [35]. The most common example is the congenital bilateral absence of the vas deferens (CBAVD), which is a cause of male infertility. Other potential CFTR-RDs include pancreatitis and rhinosinusitis. The risk of developing these conditions is not currently quantifiable, and counselling families and CFSPID individuals about the long-term risk is challenging. Whilst possibly the information may represent some benefit to the family of an early diagnosis in some cases, overall the situation raises a number of ethical issues, not least those around autonomy and non-maleficence, with the risk of over-medicalisation and iatrogenic harm. Qualitative studies of parents in this situation have demonstrated the profound negative impact this designation has on health beliefs and behaviours and the undermining of trust in health professionals [36].

There is no evidence that recognition of CFSPID improves outcomes, although some argue that it empowers families to make health decisions. In light of this, the European Neonatal Screening Working Group suggested in their best practice document that NBS protocols should aim to minimise the recognition of CFSPID infants [37]. This may be achieved by only reporting CF-causing variants in larger DNA panels and not reporting carriers or variants of varying clinical consequence. This raises the issue of autonomy in not reporting a result, even if that result is unclear and screened out by a computer (algorithm independent of human awareness). There may be an ethical argument that such selective reporting improves the performance of the programme in other regards, for example positive predictive value (PPV) and therefore this is compensated by the reduction in false positives. Consensus documents provide evidence-based guidance on the management of CFSPID with advice that minimises the medicalisation of these families [34]. Despite this, CFSPID infants are at risk of iatrogenic harm through inappropriate investigation and treatment, counter to the ethical principle of non-maleficence. 

It is too early to evaluate the impact of a CFSPID designation on the child as they grow, but there is certainly a risk of psychological morbidity if it is not handled sensitively. This will need research to characterise the impact and provide guidance. 

### 2.7. False Negative: An Affected, Undetected Infant—An infant with CF Not Detected by NBS

The outcome of newborn screening with the most potential for harm is a false negative result. The number of affected but not detected infants is a key performance measure of a screening programme. For most programmes, the commonest cause of a false negative result is an initial IRT result that is below the cut-off for further testing [32]. This is dependent on the cut-off used and the age of the baby when the dried blood sample is taken (IRT levels fall over the first weeks of life). Decreasing the IRT cut-off may improve the sensitivity, but this will be at the expense of a potential reduction in specificity [32]. A balance needs to be achieved between the improved sensitivity from lowering the cut-off and the reduction in the positive predictive value. Deciding this requires the weighing of different ethical values. Other strategies can be utilised to improve PPV and these include more extended DNA analysis, but again this can impact on the performance of other aspects of the NBS. For example, in a region with an ethnically diverse population, unusual CFTR variants may be missed and the sensitivity reduced. An approach to counter this has been the use of a “safety net”, where extremely high IRT values result in further diagnostic testing even if no CFTR mutations are recognized [1]. Whilst this strategy is employed by many programmes, again it can result in a reduction in PPV. It can be seen, therefore, that different approaches can have positive and negative impacts on sensitivity and these need to be carefully considered in the bioethical framework.

The potential negative impacts of a false negative NBS result:

- The infant may have nutritional compromise or an airway infection that may have been avoided with early recognition.
- In a region with established NBS for CF, physicians may be less likely to consider a CF diagnosis and the family may have a prolonged diagnostic journey.
- Non-Caucasian populations may be disadvantaged by the use of DNA panels designed for populations from a North European background [1].
- Parents are denied knowledge (that their child has CF) that may inform future reproductive decision making.
- A false negative result may undermine confidence in the healthcare service.

Programmes should establish robust mechanisms to collect false negative data in order to usefully evaluate the impact on the population. However, it is important that false negative results are considered in the context of other aspects of the programme and not over emphasised at the expense of other performance metrics.

## 3. The Importance of Clear Communication

Research has shown the importance of effective and open communication with relatives during the newborn screening process; for example, the reasons for persisting anxieties experienced by parents affected by a false positive result were given as uncertainty about the reliability of tests and a poor understanding of the screening and diagnostic process, showing the importance of clear and accurate communication. A study on false positive results showed that there was almost a universal perception of the newborn screening test as an “indispensable tool”; admittedly, this is for parents affected by a false positive result and therefore cannot be generalised to those affected by a false negative result, but this finding does demonstrate the importance of educating and informing relatives about the screening process [27]. Certainly, consensus guidelines highlight the importance of ensuring positive and effective communication with families, and this is seen as an imperative component of all newborn screening programmes for CF.

## 4. Achieving an Ethical Balance

There are elements of the NBS protocol that will impact on the performance of the screening programme (Table 1). These need to be considered when developing a programme in light of four main factors:

1. What is the geographical spread of the population?
2. What healthcare provision is accessible for the population?
3. What is the CFTR genetic profile of the population?
4. What is the capacity of a health system to provide clear information to parents/carers (antenatally, postnatally) and for the child as they grow into adult life?

The first question to address is: is it ethically justified to screen this population for CF? The incidence of CF in a population correlates well with the prevalence of the F508del variant. In populations with a low F508del prevalence, the incidence of CF will be low, and the ethical balance will be tilted towards not screening, as screening will result in more negative impacts (for example, a poor PPV) than positive (actual cases of CF identified). An example to consider is screening in populations from sub-Saharan Africa. Studies in the US have demonstrated higher IRT levels in black infants compared to other ethnic populations, and it is reasonable to translate those data to the majority of infants born in sub-Saharan Africa [38]. This population also has a low F508del prevalence, consistent with a low overall prevalence of CF. Screening newborns from this population will result in a disproportionate number of false positive results (poor PPV) and an increased CFSPID recognition, especially if a DNA-based protocol is used. Our ethical framework clearly does not support NBS for CF in this population at present, as the balance of limited benefit is outweighed by the significant negative impact of diagnostic uncertainty and false positive tests for many families.

The decision to screen or not to screen is also influenced by the factors above; for example, if the family are geographically accessible and the healthcare infrastructure means the family can be visited to obtain a second DBS, then it may be possible to design a programme with improved PPV that is ethically acceptable. The “standards of care” published by the ECFS suggest that with an incidence of above 1 in 7000 births, the appropriateness of NBS for CF should be considered [28].

The geographical location and the framework of healthcare provision both impact on the capability to undertake a second DBS and high-quality sweat testing. These are important considerations when determining the ethical balance of a programme (Table 1). For example, if the programme is screening a geographically dispersed population with limited access to diagnostic testing, a protocol with good PPV, ideally from a single DBS sample, would be preferred from an ethical perspective, even at the expense of reduced sensitivity. In Turkey, because of the low F508del prevalence in the population, it was decided to implement a two stage IRT-IRT protocol. In most areas of the country, this required the family to travel to a local health centre for the collection of the second DBS sample, as it was not universally possible for a healthcare professional to travel to the family home. This subsequently resulted in a number of families not attending and incomplete testing. Establishing an IRT-IRT protocol reduced some of the harms associated with the recognition of CFSPID, but the inability to complete the protocol in some cases, together with a low PPV, raises an ethical discussion to consider the balance of this approach [39]. 

It is important that a country has an adequate health infra-structure to provide a good standard of care for infants with a positive NBS result and diagnosis of CF, although there should be a recognition that, in some countries, the implementation of NBS for CF may result in the development of better services to care for children with CF. The NBS process facilitates the regional concentration of positive results and improved utilisation of resources. Finally, it is important that NBS for CF is sensitive to the cultural and religious composition of a region.

## 5. Discussion

Newborn bloodspot screening for CF is complex, and there are numerous options for the screening protocol design. Surveys by the ECFS Neonatal Screening Working Group have identified over 50 different protocols in Europe [32]. It is clear that NBS for CF fulfils the traditional and more recent criteria that are used to determine the appropriateness of screening [13]. However, in this paper we have outlined the need for a bioethical consideration of this public health intervention to better determine the most appropriate protocol for a given population. Protocols should be assessed by considering the balance between beneficence (achieving a positive impact), non-maleficence (restricting the negative impacts) and the justice and autonomy of the parents, child and wider family. It is important to characterise and acknowledge the negative outcomes recorded in this paper in order to design the most appropriate programme for a population.

To design an ethical framework for screening is a multifaceted conceptual process, one which requires consideration of the impact of an intervention on the individual, family and society, and with perspectives that encompass the biological, psychological and social facets of the intervention. The consideration of beneficence and non-maleficence must be balanced against societal justice and the rights of the individual to benefit from early recognition and treatment of their condition, as well as self-determination. 

This framework begins by addressing the ethical implications of each screening outcome, as outlined in the body of this article. This provides a guideline for those involved in the design or evaluation of newborn screening for CF and outlines the implications that ought to be considered and evaluated. The next step is to consider how to weigh the different outcomes and what ethical values should guide this, how to determine which factors should be given more significance, and how this impacts the overall outcome of the evaluation. Achieving this practice requires wide stakeholder engagement and discussion. This will allow a flexible, reflective evaluation that can be applied to programmes designed for varying populations. Once the relative ethical acceptability of the different outcomes has been determined, the next phase of the project will be using the framework to evaluate the active current screening programmes in order to determine the validity and effectiveness of the proposed framework. It is important that programmes critically review performance and adjust accordingly if they are not achieving the anticipated outcomes. Many programmes are resistant to change, but there are good examples of programmes that have adjusted cut-offs and DNA panels in light of sub-optimal performances. The national programme in France is an excellent example of a well performing programme that improved its “ethical balance” through a critical appraisal of its performance [40].

## 6. Conclusions

The complexities of newborn screening need to be considered and discussed in order to ensure the process of screening is ethical, fair, acceptable and just. The variety of potential approaches to screening introduces further complexity, and the ethical acceptability of the different outcomes will differ depending on the population screened. It is important to evaluate the process of NBS for CF and pay particular attention to the unintended consequences, including false negative results, carrier recognition and unresolved or inconclusive cases. We propose a framework for ethically evaluating NBS programmes that considers all the possible outcomes and evaluates their significance. The next step requires determining the relative acceptability of these outcomes, which will vary depending on the population; these can only be established for each population through stakeholder engagement. The framework can then be used to guide the design and evaluation of screening programmes. This framework will help guide policymakers to determine the most ethically acceptable programme, and to help identify, acknowledge and ameliorate the consequences of each outcome.

## Figures and Tables

**Table 1:** Factors to consider when designing a protocol for cystic fibrosis (CF) newborn bloodspot screening (NBS).

**Factors that will improve the sensitivity of a CF NBS programme**
A lower threshold for the first IRT value An extensive DNA panel targeted for the population screened A safety net, when a very high IRT-1 sample triggers further testing even if second tier testing (DNA or pancreatitis-associated-protein (PAP)) is negative
**Factors that will improve the positive predictive value of a CF NBS programme**
A higher IRT-1 threshold Obtaining the first DBS sample later A second IRT measurement (at day 14-21) Extended gene sequencing A second biochemical test like PAP Not screening pre-term infants Not using a safety net
**Factors that will reduce carrier recognition**
A higher IRT-1 threshold (and a later DBS sample) No DNA analysis Reduced DNA panel as a first line A second IRT sample (day 14-21) An algorithm with extended sequencing where carriers are not reported
**Factors that will reduce CFSPID recognition**
A higher IRT-1 threshold (and later sampling) A second biochemical test (PAP) Reduced DNA analysis Limiting reporting to CF causing variants after extended gene sequencing A second IRT sample (day 14-21)
